# 4,6-Bis(diphenyl­phosphino)phenoxazine (nixantphos)

**DOI:** 10.1107/S1600536808006648

**Published:** 2008-03-14

**Authors:** Thashree Marimuthu, Muhammad D. Bala, Holger B. Friedrich

**Affiliations:** a School of Chemistry, University of KwaZulu-Natal, Westville Campus, Private Bag X54001, Durban 4000, South Africa

## Abstract

The title compound, C_36_H_27_NOP_2_, has been reported as a ligand on rhodium for the catalysis of hydro­formyl­ation reactions. The key feature of the compound is the intra­molecular P⋯P distance of 4.255 (2) Å. The bond angles at the P atoms range from 99.93 (10) to 103.02 (10)°. The phenoxazine ring system is essentially planar and a non-crystallographic mirror plane through the N⋯O vector bis­ects the mol­ecule. The C—O bond lengths range from 1.388 (2) to 1.392 (2) Å and the C—N bond lengths range from 1.398 (3) to 1.403 (3) Å.

## Related literature

For related literature, see: Antonio *et al.* (1989 ▶); Claver & van Leeuwen (2000 ▶); Deprele & Montchamp (2004 ▶); van Leeuwen *et al.* (2002 ▶); Osiński *et al.* (2005 ▶); Petrassi *et al.* (2000 ▶); Ricken *et al.* (2006*a* ▶,*b* ▶,*c* ▶); Sandee *et al.* (1999 ▶, 2001 ▶); Tolman (1977 ▶); van der Veen *et al.* (2000 ▶).
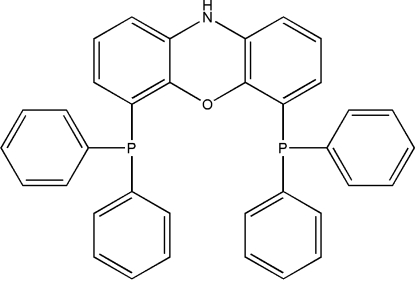


## Experimental

### 

#### Crystal data

C_36_H_27_NOP_2_
*M* *_r_* = 551.53
Triclinic, 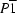

*a* = 10.4233 (3) Å
*b* = 10.9113 (3) Å
*c* = 12.9940 (4) Å
α = 104.055 (2)°
β = 102.555 (2)°
γ = 97.459 (2)°
*V* = 1373.04 (7) Å^3^
*Z* = 2
Mo *K*α radiation
μ = 0.19 mm^−1^
*T* = 173 (2) K
0.40 × 0.18 × 0.12 mm

#### Data collection

Bruker APEXII CCD area-detector diffractometer
Absorption correction: none
15968 measured reflections
5396 independent reflections
3646 reflections with *I* > 2σ(*I*)
*R*_int_ = 0.055

#### Refinement

*R*[*F* ^2^ > 2σ(*F* ^2^)] = 0.043
*wR*(*F* ^2^) = 0.105
*S* = 0.95
5396 reflections
365 parameters
H atoms treated by a mixture of independent and constrained refinement
Δρ_max_ = 0.38 e Å^−3^
Δρ_min_ = −0.29 e Å^−3^

### 

Data collection: *APEX2* (Bruker, 2005 ▶); cell refinement: *SAINT-NT* (Bruker, 2005 ▶); data reduction: *SAINT-NT*; program(s) used to solve structure: *SHELXTL* (Sheldrick, 2008 ▶); program(s) used to refine structure: *SHELXTL*; molecular graphics: *PLATON* (Spek, 2003 ▶) and *ORTEP-3* (Farrugia, 1997 ▶); software used to prepare material for publication: *SHELXTL*.

## Supplementary Material

Crystal structure: contains datablocks global, I. DOI: 10.1107/S1600536808006648/dn2322sup1.cif

Structure factors: contains datablocks I. DOI: 10.1107/S1600536808006648/dn2322Isup2.hkl

Additional supplementary materials:  crystallographic information; 3D view; checkCIF report

## Figures and Tables

**Fig. 1.**
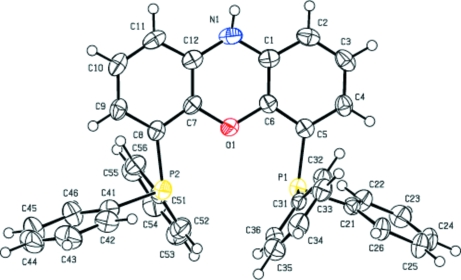
Molecular structure of the title complex with the atom labelling scheme. Ellipsoids are drawn at the 50% probability level.

## supplementary crystallographic information

### Comment

The titled compound, (1) (Fig. 1), is a xanthene based diphenylphosphine ligand. The synthesis of the ligand has been reported in literature (van der Veen *et al.*, 2000; Petrassi *et al.*, 2000; Antonio *et al.*, 1989), in addition it is commercially available and has been used extensively in synthesis and as a precursor for the synthesis of substituted bis(diphenylphoshino)phenoxazine ligands (Osiński *et al.*, 2005; Ricken *et al.*, 2006*a*,b). However, this is the first time that the crystal structure is being reported. This ligand and similar xantphos based ligands have been used on Rh as catalysts for the regioselective hydroformylation of 1-octene to octanal (Claver & van Leeuwen, 2000; van der Veen *et al.*, 2000). Moreover, (1) has been successfully immobilized on silica (Sandee *et al.*, 2001, 1999; van Leeuwen *et al.*, 2002), polystyrene (Deprele & Montchamp, 2004), and dendritic supports (Ricken *et al.*, 2006*a*).

The title compound (1) was prepared following literature procedures (Antonio *et al.*, 1989; Petrassi *et al.*, 2000) as part of our ongoing investigation of scorpionate-type ligands by the alkylation of the amine. The structural elucidation of this compound allows for the determination of important ligand factors such as the cone angle (Tolman, 1977), and the flexibility range of the natural bite angle (van der Veen *et al.*, 2000). It is also useful for studies of the coordination chemistry and catalytic applications of xantphos-type ligands. For example, the intramolecular P···P distance of 4.255 Å for (1) is similar to values reported for nixantphos-type ligands functionalized at the nitrogen (Osiński, *et al.*, 2005; Ricken *et al.*, 2006*a*,c) indicating that a functionality at N has little influence on the bite angle of the ligand.

### Experimental

The compound was synthesized *via* a three step procedure adapted from literature (Antonio *et al.*, 1989; Petrassi *et al.*, 2000; van der Veen *et al.*, 2000). Yield: 70% of yellow crystals of (1), m.p. 457–459 K. Spectroscopic analysis: ^1^H NMR (600 MHz, CDCl_3_, δ, p.p.m): 5.16 (s, 1H; NH), 5.97 (d, 2H; J(H,H) = 6.4 Hz,), 6.34 (bd, 2H;J(H,H) = 7.3 Hz,), 6.58 (t, 2H J(H,H) = 7.7 Hz), 7.17–7.23 (bs, 20H). ^13^C NMR (600 MHz, CDCl_3_, δ, p.p.m): 113.7(CH), 123.7(CH), 125.8(CH), 128.1(CH), 128.2(CH), 128.3(C), 128.3 (C), 131.3(bs,CN), 133.9(CH), 134.0(C), 136.7 (C). ^31^P NMR (600 MHz, CDCl_3_, δ, p.p.m): -19.0 MS m/*z* (%): 552.1633 (*M* + H) calculated = 552.1648 for C_36_H_27_NOP_2_ Elemental Analysis: C, 78.01; H, 4.95; N, 2.47. Found: C, 77.61; H, 4.91; N,2.41. FTIR: cm^-1^ = 3408(w), (NH), 1565(*s*), 1452(*s*), 1398(*s*), 1286, CN,1256(*m*), 1206(*m*), 1090(*m*), 766(*m*), 739(*m*), (NH), 723(*m*), 690(*s*).

### Refinement

All H atoms attached to C atoms were fixed geometrically and treated as riding with C—H = 0.95 Å and *U*_iso_(H) = 1.2*U*_eq_(C). H atom attached to nitrogen was freely refined.

### Crystal data

**Table:**

C_36_H_27_NOP_2_	*Z* = 2
*M**_r_* = 551.53	*F*_000_ = 576
Triclinic, *P*1	*D*_x_ = 1.334 Mg m^−^^3^
Hall symbol: -P 1	Melting point: 457(2) K
*a* = 10.4233 (3) Å	Mo *K*α radiation λ = 0.71073 Å
*b* = 10.9113 (3) Å	Cell parameters from 3152 reflections
*c* = 12.9940 (4) Å	θ = 2.2–25.5º
α = 104.055 (2)º	µ = 0.19 mm^−^^1^
β = 102.555 (2)º	*T* = 173 (2) K
γ = 97.459 (2)º	Triangular, yellow
*V* = 1373.04 (7) Å^3^	0.40 × 0.18 × 0.12 mm

### Data collection

**Table:**

Bruker SMART CCD area-detector diffractometer	3646 reflections with *I* > 2σ(*I*)
Radiation source: fine-focus sealed tube	*R*_int_ = 0.055
Monochromator: graphite	θ_max_ = 26.0º
*T* = 173(2) K	θ_min_ = 1.7º
φ and ω scans	*h* = −10→12
Absorption correction: none	*k* = −13→13
15968 measured reflections	*l* = −16→16
5396 independent reflections	

### Refinement

**Table:**

Refinement on *F*^2^	Secondary atom site location: difference Fourier map
Least-squares matrix: full	Hydrogen site location: inferred from neighbouring sites
*R*[*F*^2^ > 2σ(*F*^2^)] = 0.043	H atoms treated by a mixture of independent and constrained refinement
*wR*(*F*^2^) = 0.106	*w* = 1/[σ^2^(*F*_o_^2^) + (0.048*P*)^2^] where *P* = (*F*_o_^2^ + 2*F*_c_^2^)/3
*S* = 0.95	(Δ/σ)_max_ = 0.001
5396 reflections	Δρ_max_ = 0.38 e Å^−^^3^
365 parameters	Δρ_min_ = −0.29 e Å^−^^3^
Primary atom site location: structure-invariant direct methods	Extinction correction: none

### Special details

**Table:**

Geometry. All e.s.d.'s (except the e.s.d. in the dihedral angle between two l.s. planes) are estimated using the full covariance matrix. The cell e.s.d.'s are taken into account individually in the estimation of e.s.d.'s in distances, angles and torsion angles; correlations between e.s.d.'s in cell parameters are only used when they are defined by crystal symmetry. An approximate (isotropic) treatment of cell e.s.d.'s is used for estimating e.s.d.'s involving l.s. planes.
Refinement. Refinement of *F*^2^ against ALL reflections. The weighted *R*-factor *wR* and goodness of fit *S* are based on *F*^2^, conventional *R*-factors *R* are based on *F*, with *F* set to zero for negative *F*^2^. The threshold expression of *F*^2^ > σ(*F*^2^) is used only for calculating *R*-factors(gt) *etc*. and is not relevant to the choice of reflections for refinement. *R*-factors based on *F*^2^ are statistically about twice as large as those based on *F*, and *R*- factors based on ALL data will be even larger.

### Fractional atomic coordinates and isotropic or equivalent isotropic displacement parameters (Å^2^)

**Table:**

	*x*	*y*	*z*	*U*_iso_*/*U*_eq_	
C1	0.0632 (2)	0.3597 (2)	0.56267 (18)	0.0339 (5)	
C2	0.1275 (2)	0.4755 (2)	0.63818 (19)	0.0391 (6)	
H2	0.1111	0.4968	0.7089	0.047*	
C3	0.2159 (2)	0.5608 (2)	0.61113 (19)	0.0401 (6)	
H3	0.2593	0.6407	0.6633	0.048*	
C4	0.2414 (2)	0.5308 (2)	0.50922 (19)	0.0347 (5)	
H4	0.3029	0.5900	0.4921	0.042*	
C5	0.1780 (2)	0.41454 (19)	0.43100 (17)	0.0288 (5)	
C6	0.0885 (2)	0.33272 (19)	0.45990 (18)	0.0300 (5)	
C7	−0.0601 (2)	0.1287 (2)	0.40544 (18)	0.0307 (5)	
C8	−0.1125 (2)	0.0139 (2)	0.32431 (18)	0.0311 (5)	
C9	−0.1946 (2)	−0.0809 (2)	0.34914 (19)	0.0350 (5)	
H9	−0.2323	−0.1612	0.2954	0.042*	
C10	−0.2214 (2)	−0.0594 (2)	0.4502 (2)	0.0393 (6)	
H10	−0.2765	−0.1250	0.4660	0.047*	
C11	−0.1690 (2)	0.0565 (2)	0.5287 (2)	0.0395 (6)	
H11	−0.1897	0.0709	0.5978	0.047*	
C12	−0.0863 (2)	0.1525 (2)	0.50796 (19)	0.0341 (5)	
C21	0.3147 (2)	0.50031 (19)	0.28652 (17)	0.0283 (5)	
C22	0.2595 (2)	0.6054 (2)	0.2703 (2)	0.0390 (6)	
H22	0.1680	0.6049	0.2692	0.047*	
C23	0.3343 (2)	0.7100 (2)	0.2558 (2)	0.0415 (6)	
H23	0.2950	0.7818	0.2470	0.050*	
C24	0.4657 (2)	0.7113 (2)	0.25396 (19)	0.0400 (6)	
H24	0.5171	0.7829	0.2424	0.048*	
C25	0.5217 (2)	0.6081 (2)	0.2690 (2)	0.0423 (6)	
H25	0.6126	0.6084	0.2679	0.051*	
C26	0.4479 (2)	0.5037 (2)	0.28585 (19)	0.0351 (5)	
H26	0.4888	0.4335	0.2971	0.042*	
C31	0.3144 (2)	0.24846 (18)	0.31367 (17)	0.0280 (5)	
C32	0.4063 (2)	0.26320 (19)	0.41242 (18)	0.0329 (5)	
H32	0.4126	0.3345	0.4734	0.040*	
C33	0.4891 (2)	0.1757 (2)	0.4233 (2)	0.0401 (6)	
H33	0.5516	0.1867	0.4916	0.048*	
C34	0.4812 (2)	0.0733 (2)	0.3362 (2)	0.0450 (6)	
H34	0.5383	0.0131	0.3440	0.054*	
C35	0.3916 (3)	0.0568 (2)	0.2377 (2)	0.0479 (7)	
H35	0.3872	−0.0142	0.1770	0.057*	
C36	0.3075 (2)	0.1434 (2)	0.2262 (2)	0.0385 (6)	
H36	0.2445	0.1309	0.1579	0.046*	
C41	−0.1440 (2)	−0.1688 (2)	0.12171 (18)	0.0346 (5)	
C42	−0.0664 (3)	−0.2619 (2)	0.1300 (2)	0.0457 (6)	
H42	0.0234	−0.2367	0.1738	0.055*	
C43	−0.1170 (3)	−0.3900 (2)	0.0759 (2)	0.0570 (8)	
H43	−0.0627	−0.4525	0.0831	0.068*	
C44	−0.2466 (3)	−0.4270 (2)	0.0113 (2)	0.0568 (8)	
H44	−0.2820	−0.5152	−0.0263	0.068*	
C45	−0.3243 (3)	−0.3372 (2)	0.0015 (2)	0.0567 (7)	
H45	−0.4137	−0.3629	−0.0432	0.068*	
C46	−0.2737 (2)	−0.2084 (2)	0.0563 (2)	0.0465 (6)	
H46	−0.3288	−0.1467	0.0488	0.056*	
C51	−0.1711 (2)	0.09115 (19)	0.13194 (18)	0.0325 (5)	
C52	−0.1280 (2)	0.1525 (2)	0.0605 (2)	0.0459 (6)	
H52	−0.0439	0.1441	0.0455	0.055*	
C53	−0.2058 (3)	0.2257 (3)	0.0107 (2)	0.0557 (7)	
H53	−0.1746	0.2680	−0.0375	0.067*	
C54	−0.3276 (3)	0.2372 (2)	0.0307 (2)	0.0525 (7)	
H54	−0.3819	0.2858	−0.0052	0.063*	
C55	−0.3718 (3)	0.1792 (2)	0.1020 (2)	0.0453 (6)	
H55	−0.4559	0.1887	0.1166	0.054*	
C56	−0.2939 (2)	0.1070 (2)	0.15255 (19)	0.0386 (6)	
H56	−0.3249	0.0674	0.2025	0.046*	
N1	−0.0284 (2)	0.27159 (19)	0.58580 (18)	0.0414 (5)	
H1	−0.018 (3)	0.277 (3)	0.655 (2)	0.079 (11)*	
O1	0.02287 (15)	0.21948 (13)	0.37894 (12)	0.0384 (4)	
P1	0.20112 (5)	0.36083 (5)	0.29234 (5)	0.03016 (16)	
P2	−0.06220 (6)	−0.00273 (5)	0.19630 (5)	0.03407 (16)	

### Atomic displacement parameters (Å^2^)

**Table:**

	*U*^11^	*U*^22^	*U*^33^	*U*^12^	*U*^13^	*U*^23^
C1	0.0333 (13)	0.0373 (13)	0.0362 (14)	0.0149 (10)	0.0118 (11)	0.0126 (10)
C2	0.0404 (14)	0.0482 (14)	0.0305 (14)	0.0187 (11)	0.0101 (11)	0.0078 (11)
C3	0.0358 (13)	0.0364 (13)	0.0387 (15)	0.0118 (11)	0.0014 (11)	−0.0018 (11)
C4	0.0260 (12)	0.0330 (12)	0.0406 (14)	0.0056 (9)	0.0035 (10)	0.0066 (10)
C5	0.0252 (11)	0.0298 (11)	0.0323 (13)	0.0114 (9)	0.0042 (10)	0.0100 (9)
C6	0.0275 (11)	0.0285 (11)	0.0325 (13)	0.0092 (9)	0.0058 (10)	0.0057 (9)
C7	0.0253 (11)	0.0354 (12)	0.0376 (14)	0.0088 (9)	0.0108 (10)	0.0177 (10)
C8	0.0231 (11)	0.0348 (12)	0.0363 (13)	0.0068 (9)	0.0041 (10)	0.0142 (10)
C9	0.0246 (12)	0.0394 (13)	0.0404 (14)	0.0024 (9)	0.0038 (10)	0.0161 (10)
C10	0.0285 (12)	0.0452 (14)	0.0503 (16)	0.0036 (10)	0.0099 (11)	0.0267 (12)
C11	0.0325 (13)	0.0562 (16)	0.0419 (15)	0.0157 (11)	0.0179 (11)	0.0251 (12)
C12	0.0312 (12)	0.0396 (13)	0.0378 (14)	0.0139 (10)	0.0113 (11)	0.0167 (11)
C21	0.0295 (12)	0.0278 (11)	0.0261 (12)	0.0030 (9)	0.0050 (9)	0.0077 (9)
C22	0.0348 (13)	0.0386 (13)	0.0498 (16)	0.0118 (10)	0.0137 (11)	0.0188 (11)
C23	0.0503 (15)	0.0314 (13)	0.0462 (15)	0.0139 (11)	0.0113 (12)	0.0151 (11)
C24	0.0403 (14)	0.0307 (12)	0.0446 (15)	−0.0036 (10)	0.0051 (11)	0.0130 (11)
C25	0.0273 (12)	0.0406 (14)	0.0569 (17)	0.0009 (10)	0.0057 (11)	0.0174 (12)
C26	0.0293 (12)	0.0290 (12)	0.0462 (15)	0.0044 (9)	0.0051 (11)	0.0141 (10)
C31	0.0272 (11)	0.0237 (11)	0.0336 (13)	0.0002 (8)	0.0110 (10)	0.0085 (9)
C32	0.0359 (13)	0.0277 (11)	0.0355 (14)	0.0068 (9)	0.0093 (11)	0.0091 (9)
C33	0.0360 (13)	0.0389 (13)	0.0486 (16)	0.0067 (10)	0.0092 (12)	0.0201 (12)
C34	0.0409 (14)	0.0321 (13)	0.0689 (19)	0.0132 (11)	0.0205 (14)	0.0182 (12)
C35	0.0511 (16)	0.0307 (13)	0.0604 (19)	0.0103 (11)	0.0233 (14)	0.0007 (12)
C36	0.0361 (13)	0.0352 (13)	0.0388 (14)	0.0006 (10)	0.0091 (11)	0.0040 (10)
C41	0.0373 (13)	0.0336 (12)	0.0352 (14)	0.0092 (10)	0.0109 (11)	0.0112 (10)
C42	0.0488 (15)	0.0455 (15)	0.0458 (16)	0.0169 (12)	0.0128 (13)	0.0140 (12)
C43	0.080 (2)	0.0410 (16)	0.0562 (19)	0.0263 (14)	0.0215 (16)	0.0142 (13)
C44	0.088 (2)	0.0299 (14)	0.0496 (18)	0.0051 (14)	0.0203 (16)	0.0068 (12)
C45	0.0572 (17)	0.0420 (15)	0.0559 (18)	0.0010 (13)	0.0041 (14)	0.0000 (13)
C46	0.0469 (15)	0.0356 (14)	0.0483 (16)	0.0065 (11)	0.0024 (13)	0.0055 (11)
C51	0.0355 (13)	0.0271 (11)	0.0303 (13)	−0.0020 (9)	0.0055 (10)	0.0062 (9)
C52	0.0444 (15)	0.0511 (15)	0.0436 (16)	0.0021 (12)	0.0138 (12)	0.0174 (12)
C53	0.0628 (19)	0.0604 (18)	0.0529 (18)	0.0077 (14)	0.0145 (15)	0.0351 (14)
C54	0.0589 (18)	0.0488 (16)	0.0542 (18)	0.0128 (13)	0.0074 (14)	0.0275 (13)
C55	0.0464 (15)	0.0447 (14)	0.0494 (17)	0.0136 (12)	0.0121 (13)	0.0192 (12)
C56	0.0408 (14)	0.0406 (13)	0.0399 (14)	0.0094 (11)	0.0126 (11)	0.0189 (11)
N1	0.0534 (13)	0.0434 (12)	0.0354 (13)	0.0145 (10)	0.0226 (11)	0.0132 (10)
O1	0.0460 (10)	0.0326 (8)	0.0359 (9)	−0.0022 (7)	0.0180 (8)	0.0069 (7)
P1	0.0261 (3)	0.0298 (3)	0.0338 (3)	0.0032 (2)	0.0063 (3)	0.0100 (2)
P2	0.0288 (3)	0.0356 (3)	0.0365 (4)	0.0035 (2)	0.0078 (3)	0.0096 (3)

### Geometric parameters (Å, °)

**Table:**

C1—C2	1.379 (3)	C31—C36	1.386 (3)
C1—C6	1.386 (3)	C31—P1	1.836 (2)
C1—N1	1.398 (3)	C32—C33	1.379 (3)
C2—C3	1.384 (3)	C32—H32	0.9500
C2—H2	0.9500	C33—C34	1.363 (3)
C3—C4	1.377 (3)	C33—H33	0.9500
C3—H3	0.9500	C34—C35	1.366 (3)
C4—C5	1.394 (3)	C34—H34	0.9500
C4—H4	0.9500	C35—C36	1.383 (3)
C5—C6	1.381 (3)	C35—H35	0.9500
C5—P1	1.833 (2)	C36—H36	0.9500
C6—O1	1.392 (2)	C41—C46	1.381 (3)
C7—C8	1.382 (3)	C41—C42	1.387 (3)
C7—O1	1.386 (2)	C41—P2	1.826 (2)
C7—C12	1.387 (3)	C42—C43	1.377 (3)
C8—C9	1.400 (3)	C42—H42	0.9500
C8—P2	1.825 (2)	C43—C44	1.376 (4)
C9—C10	1.372 (3)	C43—H43	0.9500
C9—H9	0.9500	C44—C45	1.362 (3)
C10—C11	1.374 (3)	C44—H44	0.9500
C10—H10	0.9500	C45—C46	1.384 (3)
C11—C12	1.384 (3)	C45—H45	0.9500
C11—H11	0.9500	C46—H46	0.9500
C12—N1	1.403 (3)	C51—C52	1.384 (3)
C21—C26	1.385 (3)	C51—C56	1.388 (3)
C21—C22	1.388 (3)	C51—P2	1.828 (2)
C21—P1	1.831 (2)	C52—C53	1.380 (3)
C22—C23	1.372 (3)	C52—H52	0.9500
C22—H22	0.9500	C53—C54	1.365 (3)
C23—C24	1.374 (3)	C53—H53	0.9500
C23—H23	0.9500	C54—C55	1.366 (3)
C24—C25	1.369 (3)	C54—H54	0.9500
C24—H24	0.9500	C55—C56	1.377 (3)
C25—C26	1.380 (3)	C55—H55	0.9500
C25—H25	0.9500	C56—H56	0.9500
C26—H26	0.9500	N1—H1	0.86 (3)
C31—C32	1.384 (3)		
			
C2—C1—C6	118.3 (2)	C31—C32—H32	119.6
C2—C1—N1	122.0 (2)	C34—C33—C32	120.0 (2)
C6—C1—N1	119.7 (2)	C34—C33—H33	120.0
C1—C2—C3	120.1 (2)	C32—C33—H33	120.0
C1—C2—H2	119.9	C33—C34—C35	120.3 (2)
C3—C2—H2	119.9	C33—C34—H34	119.8
C4—C3—C2	120.6 (2)	C35—C34—H34	119.8
C4—C3—H3	119.7	C34—C35—C36	120.1 (2)
C2—C3—H3	119.7	C34—C35—H35	120.0
C3—C4—C5	120.7 (2)	C36—C35—H35	120.0
C3—C4—H4	119.7	C35—C36—C31	120.5 (2)
C5—C4—H4	119.7	C35—C36—H36	119.8
C6—C5—C4	117.2 (2)	C31—C36—H36	119.8
C6—C5—P1	116.86 (15)	C46—C41—C42	117.8 (2)
C4—C5—P1	125.91 (18)	C46—C41—P2	125.83 (17)
C5—C6—C1	123.1 (2)	C42—C41—P2	116.31 (18)
C5—C6—O1	116.04 (19)	C43—C42—C41	121.4 (2)
C1—C6—O1	120.89 (19)	C43—C42—H42	119.3
C8—C7—O1	115.78 (19)	C41—C42—H42	119.3
C8—C7—C12	122.8 (2)	C44—C43—C42	119.7 (2)
O1—C7—C12	121.38 (19)	C44—C43—H43	120.2
C7—C8—C9	117.3 (2)	C42—C43—H43	120.2
C7—C8—P2	116.80 (16)	C45—C44—C43	120.0 (2)
C9—C8—P2	125.87 (17)	C45—C44—H44	120.0
C10—C9—C8	120.8 (2)	C43—C44—H44	120.0
C10—C9—H9	119.6	C44—C45—C46	120.4 (3)
C8—C9—H9	119.6	C44—C45—H45	119.8
C9—C10—C11	120.5 (2)	C46—C45—H45	119.8
C9—C10—H10	119.7	C41—C46—C45	120.8 (2)
C11—C10—H10	119.7	C41—C46—H46	119.6
C10—C11—C12	120.6 (2)	C45—C46—H46	119.6
C10—C11—H11	119.7	C52—C51—C56	117.8 (2)
C12—C11—H11	119.7	C52—C51—P2	118.61 (18)
C11—C12—C7	118.0 (2)	C56—C51—P2	123.61 (18)
C11—C12—N1	122.9 (2)	C53—C52—C51	120.9 (2)
C7—C12—N1	119.1 (2)	C53—C52—H52	119.6
C26—C21—C22	117.61 (19)	C51—C52—H52	119.6
C26—C21—P1	124.86 (16)	C54—C53—C52	120.0 (2)
C22—C21—P1	117.19 (16)	C54—C53—H53	120.0
C23—C22—C21	121.4 (2)	C52—C53—H53	120.0
C23—C22—H22	119.3	C53—C54—C55	120.4 (2)
C21—C22—H22	119.3	C53—C54—H54	119.8
C22—C23—C24	120.3 (2)	C55—C54—H54	119.8
C22—C23—H23	119.8	C54—C55—C56	119.7 (2)
C24—C23—H23	119.8	C54—C55—H55	120.1
C25—C24—C23	119.1 (2)	C56—C55—H55	120.1
C25—C24—H24	120.4	C55—C56—C51	121.2 (2)
C23—C24—H24	120.4	C55—C56—H56	119.4
C24—C25—C26	120.8 (2)	C51—C56—H56	119.4
C24—C25—H25	119.6	C1—N1—C12	119.7 (2)
C26—C25—H25	119.6	C1—N1—H1	115 (2)
C25—C26—C21	120.7 (2)	C12—N1—H1	119.0 (19)
C25—C26—H26	119.7	C7—O1—C6	118.82 (17)
C21—C26—H26	119.7	C21—P1—C5	101.96 (10)
C32—C31—C36	118.29 (19)	C21—P1—C31	102.20 (9)
C32—C31—P1	123.48 (15)	C5—P1—C31	99.98 (9)
C36—C31—P1	118.23 (17)	C8—P2—C41	100.81 (10)
C33—C32—C31	120.8 (2)	C8—P2—C51	99.93 (10)
C33—C32—H32	119.6	C41—P2—C51	103.02 (10)
			
C6—C1—C2—C3	−0.8 (3)	C41—C42—C43—C44	0.6 (4)
N1—C1—C2—C3	−178.9 (2)	C42—C43—C44—C45	−0.2 (4)
C1—C2—C3—C4	−0.5 (3)	C43—C44—C45—C46	−0.1 (4)
C2—C3—C4—C5	0.5 (3)	C42—C41—C46—C45	0.4 (4)
C3—C4—C5—C6	0.7 (3)	P2—C41—C46—C45	178.5 (2)
C3—C4—C5—P1	179.98 (16)	C44—C45—C46—C41	0.0 (4)
C4—C5—C6—C1	−2.0 (3)	C56—C51—C52—C53	−0.7 (3)
P1—C5—C6—C1	178.60 (16)	P2—C51—C52—C53	−179.29 (19)
C4—C5—C6—O1	177.72 (17)	C51—C52—C53—C54	−0.7 (4)
P1—C5—C6—O1	−1.7 (2)	C52—C53—C54—C55	1.6 (4)
C2—C1—C6—C5	2.1 (3)	C53—C54—C55—C56	−1.0 (4)
N1—C1—C6—C5	−179.71 (19)	C54—C55—C56—C51	−0.4 (4)
C2—C1—C6—O1	−177.62 (18)	C52—C51—C56—C55	1.3 (3)
N1—C1—C6—O1	0.6 (3)	P2—C51—C56—C55	179.79 (18)
O1—C7—C8—C9	178.75 (17)	C2—C1—N1—C12	−177.0 (2)
C12—C7—C8—C9	−0.3 (3)	C6—C1—N1—C12	4.9 (3)
O1—C7—C8—P2	1.3 (2)	C11—C12—N1—C1	174.5 (2)
C12—C7—C8—P2	−177.74 (16)	C7—C12—N1—C1	−5.5 (3)
C7—C8—C9—C10	0.1 (3)	C8—C7—O1—C6	−174.40 (17)
P2—C8—C9—C10	177.27 (16)	C12—C7—O1—C6	4.7 (3)
C8—C9—C10—C11	0.7 (3)	C5—C6—O1—C7	174.95 (17)
C9—C10—C11—C12	−1.3 (3)	C1—C6—O1—C7	−5.3 (3)
C10—C11—C12—C7	1.0 (3)	C26—C21—P1—C5	109.83 (19)
C10—C11—C12—N1	−178.9 (2)	C22—C21—P1—C5	−77.11 (18)
C8—C7—C12—C11	−0.2 (3)	C26—C21—P1—C31	6.7 (2)
O1—C7—C12—C11	−179.27 (18)	C22—C21—P1—C31	179.77 (17)
C8—C7—C12—N1	179.68 (19)	C6—C5—P1—C21	175.90 (15)
O1—C7—C12—N1	0.7 (3)	C4—C5—P1—C21	−3.4 (2)
C26—C21—C22—C23	−1.0 (3)	C6—C5—P1—C31	−79.23 (17)
P1—C21—C22—C23	−174.60 (19)	C4—C5—P1—C31	101.45 (18)
C21—C22—C23—C24	1.9 (4)	C32—C31—P1—C21	73.74 (19)
C22—C23—C24—C25	−1.3 (4)	C36—C31—P1—C21	−106.18 (18)
C23—C24—C25—C26	0.0 (4)	C32—C31—P1—C5	−30.94 (19)
C24—C25—C26—C21	0.8 (4)	C36—C31—P1—C5	149.14 (17)
C22—C21—C26—C25	−0.3 (3)	C7—C8—P2—C41	176.03 (16)
P1—C21—C26—C25	172.73 (18)	C9—C8—P2—C41	−1.1 (2)
C36—C31—C32—C33	0.1 (3)	C7—C8—P2—C51	−78.53 (17)
P1—C31—C32—C33	−179.82 (17)	C9—C8—P2—C51	104.31 (19)
C31—C32—C33—C34	0.2 (3)	C46—C41—P2—C8	87.2 (2)
C32—C33—C34—C35	0.1 (4)	C42—C41—P2—C8	−94.69 (19)
C33—C34—C35—C36	−0.6 (4)	C46—C41—P2—C51	−15.8 (2)
C34—C35—C36—C31	0.9 (4)	C42—C41—P2—C51	162.34 (18)
C32—C31—C36—C35	−0.7 (3)	C52—C51—P2—C8	151.05 (18)
P1—C31—C36—C35	179.27 (18)	C56—C51—P2—C8	−27.4 (2)
C46—C41—C42—C43	−0.7 (4)	C52—C51—P2—C41	−105.31 (19)
P2—C41—C42—C43	−179.0 (2)	C56—C51—P2—C41	76.2 (2)
